# MicroRNA-22 functions as a tumor suppressor by targeting SIRT1 in renal cell carcinoma

**DOI:** 10.3892/or.2021.8194

**Published:** 2021-09-23

**Authors:** Shoulin Zhang, Dongmei Zhang, Chunguang Yi, Yinping Wang, Hongan Wang, Jian Wang

Oncol Rep 35: 559-567, 2016; DOI: 10.3892/or.2015.4333

Following the publication of this paper, the authors have contacted the Editorial Office to explain that they are unable to reproduce the results of various of their experiments after having repeated some of them, and consequently they wish to retract the paper.owing to a lack of confidence in the presented data. It was also drawn to the attention of the Office that certain of the tumor images shown in Fig. 6A were strikingly similar to data appearing in different form in other articles by different authors. Considering all these points, the Editor of *Oncology Reports* is in agreement that this paper should be retracted from the Journal.

The authors all agree with the decision to retract this paper. The Editor apologizes to the readership for any inconvenience caused.

